# Effect of Salts on the Conformational Dynamics of the Cytochrome P450 OleP

**DOI:** 10.3390/molecules28020832

**Published:** 2023-01-13

**Authors:** Maria Laura De Sciscio, Alessandro Nicola Nardi, Giacomo Parisi, Giovanni Bulfaro, Antonella Costanzo, Elena Gugole, Cécile Exertier, Ida Freda, Carmelinda Savino, Beatrice Vallone, Linda Celeste Montemiglio, Marco D’Abramo

**Affiliations:** 1 Department of Chemistry, University of Rome, Sapienza, P.le A. Moro 5, 00185 Rome, Italy; 2 Center for Life Nano & Neuro-Science, Fondazione Istituto Italiano di Tecnologia, IIT, 00185 Rome, Italy; 3 Department of Biochemical Sciences “A. Rossi Fanelli”, University of Rome, Sapienza, P.le A. Moro 5, 00185 Rome, Italy; 4 Takis Biotech, Via di Castel Romano 100, 00128 Rome, Italy; 5 Institute of Molecular Biology and Pathology, CNR c/o Department of Biochemical Sciences “A. Rossi Fanelli”, University of Rome, Sapienza, P.le A. Moro 5, 00185 Rome, Italy

**Keywords:** cytochrome, ion tethering, equilibrium binding, molecular dynamics

## Abstract

Cytochrome P450 OleP catalytic activity is strongly influenced by its structural dynamic conformational behavior. Here, we combine equilibrium-binding experiments with all-atom molecular dynamics simulations to clarify how different environments affect OleP conformational equilibrium between the open and the closed—catalytic competent—forms. Our data clearly show that at high-ionic strength conditions, the closed form is favored, and, very interestingly, different mechanisms, depending on the chemistry of the cations, can be used to rationalize such an effect.

## 1. Introduction

OleP (CYP107D1) is the cytochrome P450 from *Streptomyces antibioticus* involved in the later steps of oleandomycin biosynthesis [1,2,3]. This enzyme catalyzes the regio- and stereospecific epoxidation of the C8-C8a of the macrolactone ring of two metabolic intermediates, namely the aglycone 8.8a-deoxyoleandolide (DEO) and the monoglycosylated L-olivosyl-8.8a deoxyoleandolide (L-O-DEO) [4].

**Figure 1:**
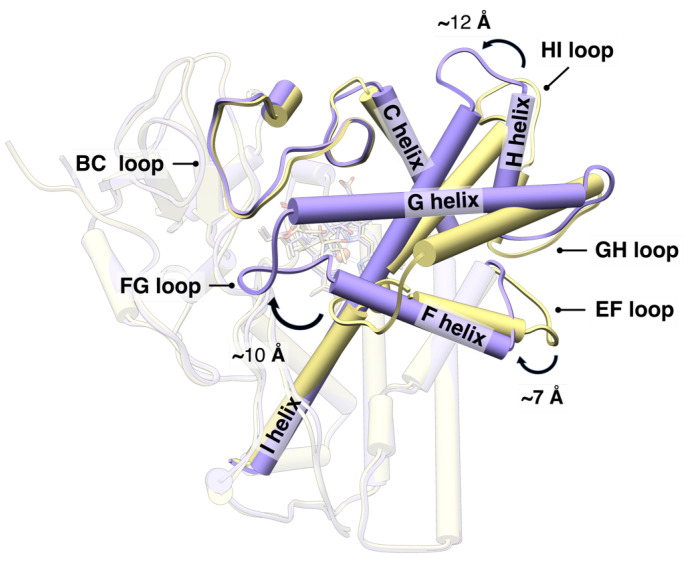
Conformational change of the substrate-bound OleP. Top view of the open (yellow) and closed (purple) structures of OleP-6DEB (6-deoxyerythronolide B, abbreviated in 6DEB) complex (pdb id 5MNV and 5MNS, respectively [5]) superposed. The secondary structure elements mainly involved in the conformational change are labelled and highlighted. Helices are represented as cylinders. Arrows indicate the direction of the open-to-closed transition. The 6DEB and heme are represented in transparency as sticks.

Substrate binding to OleP determines the accumulation of a heterogeneous ensemble of substrate-bound OleP forms [5]. Crystallographic and binding kinetic data revealed that the substrate-binding reaction follows an allosteric induced-fit mechanism, whereby an initial fast phase of substrate entrance into the heme pocket is followed by a slow phase of conformational transition. The large open-to-closed structural change experienced by the substrate-bound OleP involves specific secondary structural elements that define the active site (i.e., the F and G helices, the FG, HI, and BC loops and the internal I helix) that rearrange, enabling the enzyme to achieve the catalytically competent structure with a productive repositioning of the substrate (see Figure 1) [5,6,7]. In fact, the position adopted by the substrate promotes the displacement of the distal heme-iron coordinating water molecule, leaving a five-coordinate high-spin heme, which is ready for the first step of electron transfer only when the active site is closed [8,9]. In this state, the internal and substrate sensitive I helix bends and breaks the helical geometry of a central turn, forming the typical cleft responsible for the catalytic proton relay network [5,7,10]. Direct or indirect contacts established between the substrate and residues at the N-terminus of the I helix trigger the movement of the F/G unit of OleP, contributing to the overall closure efficiency. As a result, the external solvent accessibility and active site hydration are minimized, thus limiting the occurrence of detrimental reactions and the production of reactive oxygen species during catalysis (see Figure 1).

Equilibrium-binding experiments and crystallographic analysis previously reported have shown that the conformational dynamics of the substrate-bound OleP can be altered by variations of the ionic strength conditions. More specifically, a high-ionic strength environment (I ≥ 4 M) favors the full accumulation of the closed conformer in crystals, whereas in solution, an increase of ionic strength (up to I ≈ 3.5 M) causes a transition of the absorption spectra toward the high-spin state in the spin equilibrium [5,7,11]. Therefore, increasing the ionic strength shifts the equilibrium toward the high-spin closed form of the substrate-bound OleP, which is the less populated conformer in physiological conditions (I ≈ 0.2 M) [5,7,11]. The way by which ionic strength influences the structural change of the substrate-OleP complex and whether the effect may be enhanced by specific cations remains unknown.

To address these questions, in this study the contribution of ionic strength and of different cations to the open-to-closed conformational transition of the substrate-bound OleP were examined by means of equilibrium-binding experiments and molecular dynamics simulations by using the aglycone substrate analogue 6-deoxyerythronolide B (6DEB). This compound, which was experimentally demonstrated to behave as substrate for OleP, only differs from the DEO because of the one extra carbon atom of the alkyl substituent group at the C13 [5,7].

Our results provide a description of the effect of salt/ionic strength on the conformational dynamics of the P450 OleP, unveiling the role of active site dehydration in the structural transition experienced by the substrate-bound protein. Furthermore, by taking advantage of the structures of the OleP-6DEB complex previously obtained in different ionic strength conditions, we propose a plausible role of specific intramolecular electrostatic interactions in the control of this mechanism.

## 2. Materials and Methods

### 2.1. Chemicals

Dimethyl sulfoxide (DMSO) was purchased from Millipore Merck Sigma (Burlington, MA, USA). The substrate analogue 6-deoxyerythronolide B (6DEB) was kindly supplied by Barrie Wilkinson and Rachel Lill (Biotica Technology Ltd., Cambridge, UK). The stock solution of 6DEB was prepared at a concentration of 26 mM in DMSO.

### 2.2. Equilibrium-Binding Analysis

To monitor the effect of specific cations on the binding properties of OleP to 6DEB, ≈2 µM protein was titrated with an increasing concentration of ligand, ranging from 0 to 90 µM, in a final volume of 750 µL of 50 mM 4-(2-hydroxyethyl)-1-piperazineethanesulfonic acid (Hepes), pH 7.5, at 298 K. The concentration of salts selected for this study was adjusted to keep the ionic strength at 0.5 M in all the experiments. The final percentage of DMSO was kept below 1%. The typical Type I absorption change of the Soret peak from 419 to 388 nm induced by 6DEB binding was followed by collecting UV-visible spectra (200–800 nm) after each substrate addition, subtracting the appropriate blank. The absorbance intensities at 419 and 388 nm were plotted against the logarithm of total [6DEB]. A nonlinear regression analysis was applied to the experimental data to estimate the binding affinity constant, $K_{D}$, of OleP to 6DEB by using the hyperbolic equation

(1) $$\Delta AU_{obs}=\Delta AU_{max}\left[L\right]/\left(K_{D}+\left[L\right]\right),$$

where $\Delta AU_{obs}$ is the absorbance difference, $\Delta AU_{max}$ is the maximum absorbance difference extrapolated to infinite ligand concentration, and [*L*] is the ligand analytical concentration. The $K_{D}$ was estimated with Kaleidagraph software (Synergy Software, Reading, PA, USA). All data were also globally fitted with Prism (GraphPad Software, La Jolla, CA, USA). The percentage of high spin was calculated according to Jung’s procedure [12]. All spectral determinations were made by using a HP-8453 spectrophotometer equipped with a Peltier temperature control accessory (HP-S9090A).

### 2.3. Molecular Dynamics Simulations

The molecular dynamics (MD) simulations of the open (monomer A) and closed form (monomer C) of P450 OleP bound to the 6-deoxyerythronolide B (6DEB) substrate—pdb id 5MNV [5]—in low (0.2 M) and high (3.5 M) salt (NaCl) concentrations were conducted by using the Gromacs 2022 software package [13]. An additional set of MD simulations of the open form of substrate-bound P450 OleP in low (0.07 M) and high (1.17 M) ${\mathrm{MgCl}}_{2}$ salt concentrations were performed. These concentrations correspond to an ionic strength of 0.2 and 3.5 M, respectively.

NaCl and ${\mathrm{MgCl}}_{2}$ were chosen as representative salts to study the effect of mono- and divalent cations. The force field used is CHARMM36 [14] and for the parametrization of 6DEB, CGenFF [15] was used (see Supplementary Materials for details). The (open/closed) structure of P450 OleP was centered in a cubic box and solvated by using the TIP3P [16] water model. A suitable number of ions (${\mathrm{Na}}^{+}$ or ${\mathrm{Mg}}^{2+}$ and ${\mathrm{Cl}}^{-}$) have been inserted in the simulation box, each replacing a water molecule, to reach the correct value of ionic strength. An energy minimization step was performed by using the steepest descent algorithm without position restraints. After the minimization, a series of equilibration steps, lasting 50 ps with a timestep of 2 fs, were performed in the NVT ensemble: after each run, we checked the pressure of the system and tuned the size of the box in order to reach the correct value of the density [17]. After the equilibration steps, each system has been simulated for 500 ns at fixed temperature and volume. The temperature was kept constant at 300 K by using the V-rescale thermostat [18] by using a $\tau_{T}$ of 0.1 ps. The electrostatic interactions were calculated by using the particle mesh Ewald method [19,20] with a cutoff of 1.2 nm. A cutoff of 1.2 nm was used for the van der Waals interactions.

### 2.4. Structural Analysis

The structural analysis of the P450 OleP structures as sampled by molecular dynamics simulations at different ionic strength conditions was performed. Such analyses were based on (i) principal component analysis (PCA), (ii) Euclidean distances between key residues to quantify the extent of the closure of the structure, (iii) characterization of the protein hydration shell, and (iv) of specific local interactions.

The PCA was applied to the MD trajectories to obtain the essential subspace [21]. This means, briefly, that the *N*x*N* covariance matrix of the atomic positions is built from the MD simulation on a selected group of *N* atoms (usually C-alpha, as in the present case) and diagonalized. From the diagonalization, a set of eigenvectors and the corresponding eigenvalues are obtained. The eigenvectors represent the directions of the principal motion of the system and, therefore, they are used to describe the essential protein modes. By such an approach, it is possible to represent the protein dynamics in a reduced (essential) subspace—defined by the first few eigenvectors—which approximate well the overall molecular motions.

The root mean square fluctuation (RMSF) was used to identify the regions of the protein that are responsible for the open–close transition and/or the most fluctuating regions. In order to follow the open-to-closed conformational transition in P450 OleP induced by the increase in the ionic strength, we monitored the changes in the Euclidean distance between residue P85, located in the BC-loop, and residue T185 belonging to the G-helix.

To characterize the solvent density around the protein, we used the approximation of treating the protein molecule as an ellipsoid defined, at each MD frame, by the eigenvectors and eigenvalues of the 3 × 3 geometrical covariance matrix of the atomic Cartesian coordinates, as described in previous papers [17,22]. The directions of the instantaneous protein ellipsoid axes are identified by the three eigenvectors of the geometrical covariance matrix with the corresponding lengths provided proportionally to the square root of the eigenvalues (e.g., considering a Gaussian atomic positional distribution along each eigenvector, the semiaxis $a_{i}=2\sqrt{\lambda_{i}}$ with i=1,2,3 and $\lambda_{i}$ the eigenvalue of the *i*-th eigenvector). We then considered a set of ellipsoidal layers around the protein defined by ellipsoids with increasing semiaxes $a_{i}\left(n\right)=a_{i}+n\cdot \delta_{i}$ with a normalized increment delta defined by $\delta_{i}=0.03$ nm times the *i*-th eigenvalue divided by the maximum eigenvalue of the set. By calculating at each MD frame the instantaneous water density within each layer (disregarding the possible presence of protein atoms and/or ions) and averaging over the MD trajectory, we obtained the solvent density profile around the protein within consecutive ellipsoids of increasing volume. This analysis was also performed on negative values of *n* to obtain information on the solvent accessibility in the protein interior (i.e., in ellipsoidal inner protein layers).

To evaluate the strength of salt bridges we calculated the distance, along the MD trajectories, between interacting polar side-chain atoms; for each couple of residues, the reported distance is the mean values of the single atom-pairs distances (e.g., for carboxyl-guanidinio group interaction, the final value is the mean of the distance between one carboxyl oxygen and one amine nitrogen—first atom pair—and the other carboxyl oxygen-amine nitrogen).

## 3. Results

### 3.1. Divalent Cations Enhance the High-Spin State Subpopulation of the OleP-6DEB Complex

Equilibrium-binding experiments and X-ray structural analysis have shown that ionic strength alters the equilibrium between low- and high-spin populations of OleP bound to 6DEB: as ionic strength increases, the high-spin-state complex is stabilized, by enhancing OleP closure which allows the aglycone substrate to displace the sixth coordinating water molecule [5,7,11].

**Figure 2:**
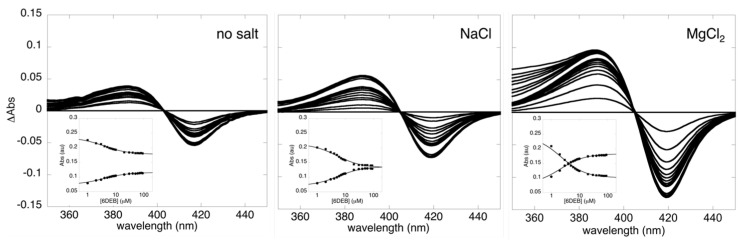
Divalent cations enhance the high-spin state shift induced by substrate binding to OleP. Equilibrium titrations of OleP with 6DEB in the absence (no salt) and in the presence of NaCl and MgCl_2_ (I = 0.5 M). Different spectral changes observed upon substrate binding are shown. Insets report the absorbance intensities of OleP monitored at 419 nm (full squares) and at 388 nm (full dots) as a function of the logarithm of total 6DEB concentration. In the experiments, the protein has been used at a constant concentration in 50 mM Hepes, pH 7.5, at 298 K. Solid lines are the best fit to a hyperbolic function.

To understand how variations in ionic strength elicit structural changes in the substratebound form of OleP, we first questioned if a contribution to this phenomenon could also arise from the presence of specific cations. Therefore, the influence of mono- and divalent cations on the spin equilibrium displayed by OleP upon substrate binding was analysed. Equilibrium-binding experiments were performed at 298 K by measuring the absorbance spectra of OleP at varied 6DEB concentrations to monitor the spin state shift of the heme iron that accompanies ligand binding to the P450 distal pocket [23]. Experiments were recorded both in the absence and in the presence of different salts, at a constant ionic strength (0.5 M). In all conditions explored, the binding of 6DEB induces the type I spectral transition typical of P450 substrates, resulting in a shift of the Soret peak from 419 to 388 nm (Figure 2 and Figure S3). The observed binding profile was satisfactorily fitted to a hyperbolic function as expected from a simple two-state equilibrium binding (Figure 2, insets). The results are summarized in Table 1, which also reports the high-spin content calculated in the absence of substrate and in the presence of saturating concentration of 6DEB.

In all examined cases, we observed only a marginal effect of salts on OleP affinity to 6DEB, the maximum difference in the estimated binding free energy being about 0.5 kcal/mol, which corresponds to the rupture/formation of a single van der Waals contact [24]. Conversely, marked differences were observed by comparing the amplitude of the spectral transitions recorded in the absence and in the presence of different salts, which accounts for the population of the enzyme that is converted to the high-spin state upon 6DEB binding [25]. At saturating concentration of 6DEB, the substrate-bound protein with no salt present is ∼46% high spin, which is consistent with a partial displacement of the sixth coordinating water molecule from the heme-iron. The presence of higher ionic strength increases the high-spin content in all salt conditions explored. Remarkably, a more pronounced spin-shift to the high state is observed in the presence of salts of divalent cations (∼70%).

### 3.2. Structural Dynamic Analysis

To gain insights into the structural dynamics behavior of OleP-6DEB complex in aqueous solution at different ionic strength conditions, we performed two sets of MD simulations of the complex, in the open conformation in water, differing in the type of salt used as a buffer. To identify the effect of the ion valence on the open–close equilibrium, the first set of simulations were conducted by using NaCl as a buffer at 0.2 and 3.5 M (corresponding to I = 0.2 and 3.5 M, respectively). The second set of simulations was conducted by using MgCl_2_ as a buffer at 0.07 and 1.17 M (corresponding to I = 0.2 and 3.5 M, respectively).

Additional simulations of the complex, in the closed conformation, in low- (0.2 M) and high- (3.5 M) ionic strength conditions by using NaCl, were performed to corroborate our findings. For more detail on the molecular dynamics simulations, see the Section 2.

#### 3.2.1. Principal Component Analysis

**Figure 3:**
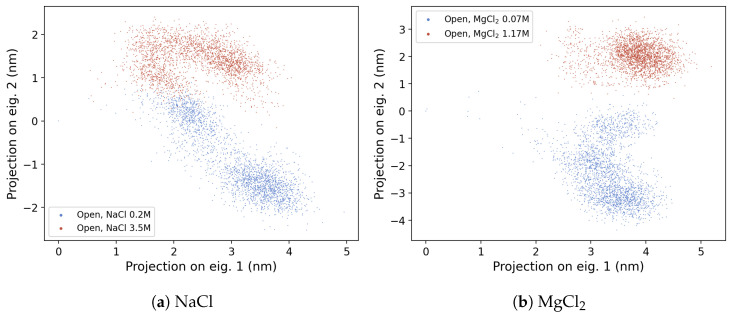
Projection of the concatenated MD trajectories of the open form of OleP-6DEB in NaCl and MgCl_2_ buffer. In both cases, the frames corresponding to the low- and high-ionic strength conditions are reported in blue and red, respectively.

The PCA performed on the concatenated trajectories of the OleP-6DEB complex in aqueous solution at different ionic strength conditions, with NaCl or MgCl_2_ as a buffer, allows us to characterize the effect of the ionic conditions on the protein conformational dynamics. In fact, in either NaCl or MgCl_2_ buffer, the trajectories at low-ionic strength conditions explore different regions of the essential subspace (represents more than 60% of the total variance) with respect to the corresponding trajectories simulated at high ionic strength, as reported in Figure 3.

**Figure 4:**
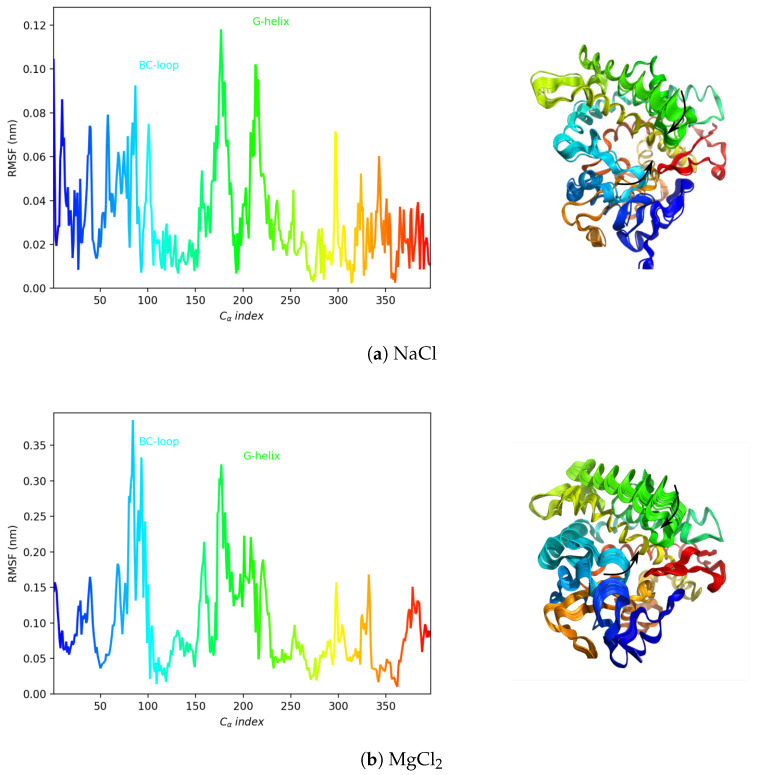
Root mean square fluctuations of the $C_{\alpha }$ atoms of the trajectories (in NaCl and MgCl_2_ buffer) projected on the first eigenvector. The $C_{\alpha }$ indexing starts from 1 for the N-terminal residue (**left**). Fifty superimposed frames of the projected trajectory onto the first eigenvector. The black arrows indicate G-helix and BC-loop closure (**right**).

To characterize the most fluctuating protein regions, the root mean square fluctuations (RMSF) of the $C_{\alpha }$ atom positions were calculated on the projected trajectory onto the first eigenvector (see Figure 4). It was found that, along this eigenvector, the relevant motion mainly describes the movement of the G-helix and BC-loop, as indicated by the back arrows in Figure 4, in both the NaCl and MgCl_2_ buffer.

**Figure 5:**
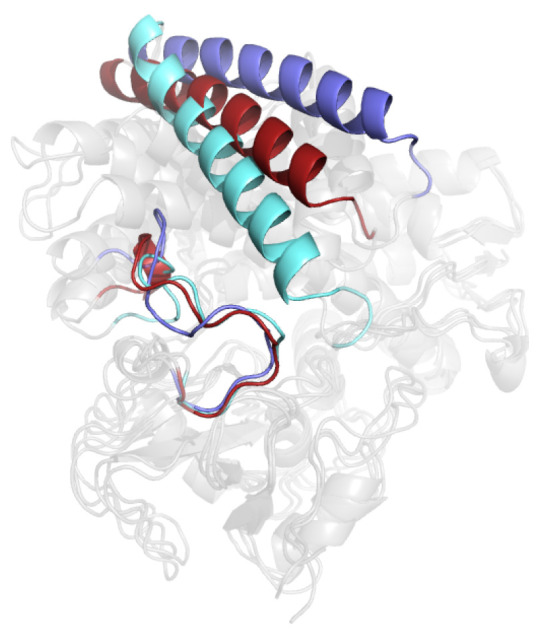
MD representative structures of the open form in high- (red and grey) and low- (blue and grey) ionic strength condition (in NaCl buffer) aligned over the crystallographic structure of the closed form of P450 OleP (cyan and grey).

In Figure 5, we report a comparison between MD representative structures of the open form of OleP-6DEB in both low- and high-ionic strength conditions in NaCl buffer and the crystallographic structure of the closed form.

This structural comparison shows that although within the MD simulation timescale the trajectories do not sample conformations typical of the closed crystallographic structure, a clear trend toward close-like conformations emerges in response to the ionic strength increase.

#### 3.2.2. G-Helix–BC-Loop Distance

**Figure 6:**
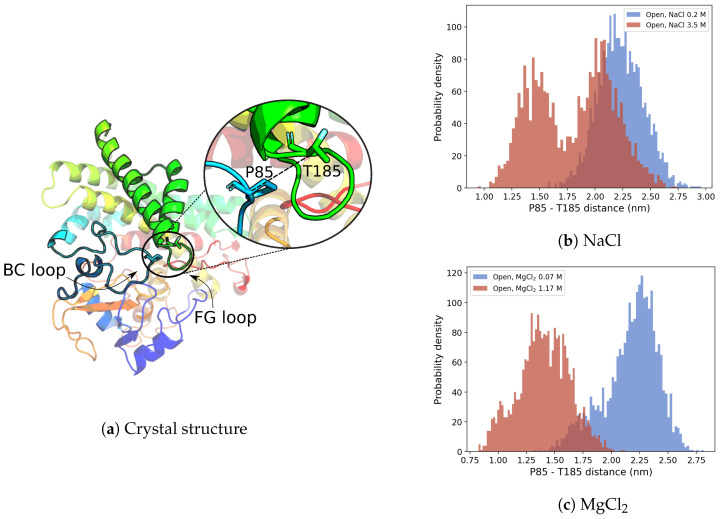
P85–T185 distance used as the observable of open-to-closed conformational transition. (**a**) P85–T185 residues, shown in sticks, their equilibrium distance (dotted line) and position within closed-form OleP crystal structure (pdb id 5MNV, C monomer [5]). (**b**,**c**) P85–T185 distance distribution along the MD trajectory of the P450 OleP in high- (red) and low- (blue) ionic strength conditions.

To measure the extent of the closure process, the distance between the residues P85 (on the BC-loop) and T185 (on the G-helix (Figure 6a), the relative positions of which experience the largest variation in the transition from the open to the closed structures) was calculated along the MD trajectories of the open conformation in both high- and low-ionic strength conditions (see Figure 6b,c). According to the closed-form OleP crystal structure [5] and MD simulation (see Figure S4), the P85–T185 distance is below 1.0 nm.

In the low-ionic strength condition in the NaCl buffer, the distribution of P85–T185 distance is unimodal with the maximum located at ≃2.2 nm. On the other hand, at high ionic strength, the distribution is bimodal and displays two maxima, one at ≃2.0 nm (the same value observed in the low ionic strength), and one at considerably lower distances, i.e., ≃1.5 nm, associated with closed conformations.

On the other hand, in MgCl_2_ buffer, at high-ionic strength condition (1.17 M), the P85–T185 distribution is shifted to lower values with respect to the NaCl buffer. In this case, the maximum at ≃ 2.0 nm—typical of the open conformations—disappears. Interestingly, also in low-ionic strength conditions ([MgCl_2_] = 0.07 M), a left tail extending up to ≃1.5 nm compatible with closed-like conformations appears (see Figure 6c).

This finding is consistent with the experimental observation that high ionic strength favors closed-like conformations of the OleP-6DEB complex [7,11].

#### 3.2.3. Protein Hydration Shell and Salt Bridges

In the previous sections, we have shown that an increase in NaCl or MgCl_2_ concentrations leads to a population shift toward closed or closed-like conformations of the complex, as observed by in vitro and in silico studies. In this section, we provide an explanation for this phenomenon in terms of hydration and protein–ion interactions as derived from MD trajectory analysis.

**Figure 7:**
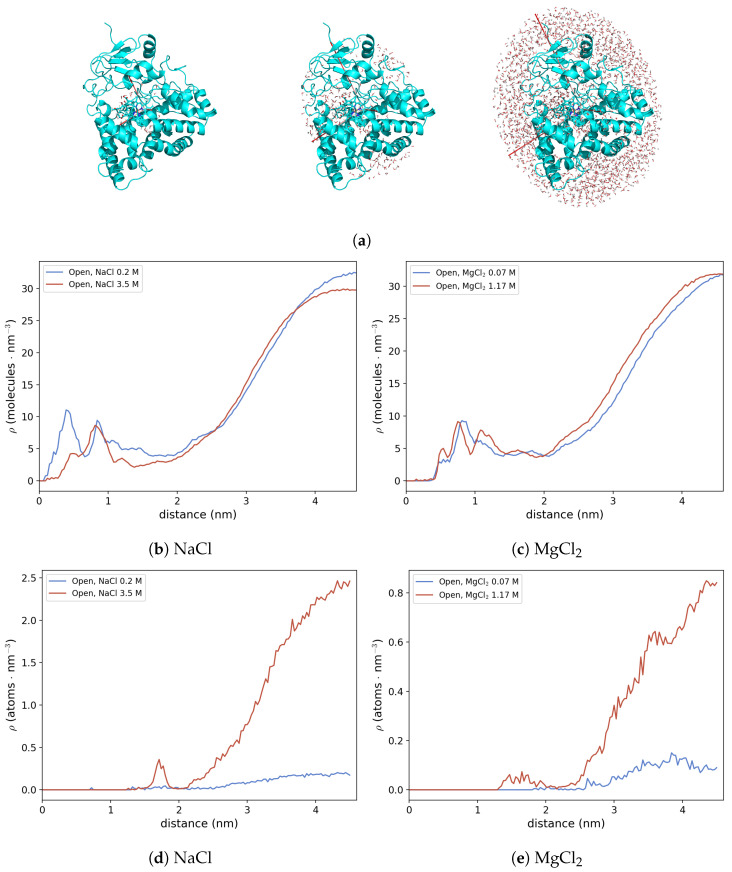
(**a**) OleP-6DEB complex structure as provided by a frame sampled by MD simulation (cyan), semiaxes of the representative ellipsoids (red) of increasing size (from left to right). The water molecules within the protein ellipsoid are represented as wires; (**b**,**c**) local solvent density (ρ, expressed as the number of solvent molecules per nm^3^) as a function of the distance, along the major ellipsoid semiaxis, from the geometrical centre of the protein. (**d**,**e**) Local ions’ density (ρ, expressed as the number of ion atoms per nm^3^) as a function of the distance, along the major ellipsoid semi-axis, from the geometrical centre of the protein.

MD simulations indicate that the active site of OleP in the open conformation is partially exposed to the solvent and that a continuous exchange of water molecules, and potentially ions, between the bulk and the active site and/or regions near the active site takes place. In order to investigate the hydration structure of the inner part of the OleP-6DEB complex and of its surface as well as the distribution of the cations around the complex, we applied a model presented in previous works to describe protein hydration shell [17,22]. Briefly, the model considers the protein as an ellipsoid described by three semiaxes given by the diagonalization of the geometrical covariance matrix, being the length of these axes proportional to their corresponding eigenvalues ($a_{i}=2\sqrt{\lambda_{i}}$ with i=1,2,3 and $\lambda_{i}$ the eigenvalue of the *i*-th eigenvector). As an example, in Figure 7a, we report the ellipsoid (that best fit the protein in that specific conformation) corresponding to one frame of the MD trajectory of the open conformation at high ionic strength. Given this ellipsoid, it is possible to calculate the local density of solvent molecules around the protein, i.e., in each direction along the semiaxis, at each frame of the MD simulation.

In the present work, we considered concentric ellipsoids defined by semiaxes of different sizes: at each frame of the MD simulations we calculate the semiaxes’ directions of the ellipsoid that best fit the OleP-6DEB complex. Starting from the geometric center of the protein, we started resizing the ellipsoid by increasing the length of the semiaxes proportional to their eigenvalue. For the sake of clarity, three ellipsoids of increasing size—representing the active site (the smallest ellipsoid, left), the OleP inner region (middle), and the protein/first solvation layer (the largest ellipsoid, right)—are reported in Figure 7a. Then, we calculated solvent and ions’ local density in the volume between the surfaces of two concentric ellipsoids, obtaining the profiles reported in Figure 7b–e.

This analysis clearly shows that the local density of solvent molecules around the active site (which corresponds to the region between 0 and 1.5 nm along the major ellipsoid semiaxis) is higher in low-ionic strength conditions in NaCl buffer. That is, the response of the system to salt increase is to expel water molecules from the protein interior, leading to a population shift towards closed-like conformations. Importantly, the two MD simulations at different NaCl concentrations were started from very similar solvation conditions.

On the other hand, in MgCl_2_ buffer, such a mechanism is not observed. In fact, at both 0.07 M and 1.17 M, the water density profiles along the ellipsoid semi-axis do not show significant differences. Therefore, in the presence of MgCl_2_, we investigated in detail whether some of the common structural observables used to discriminate between open and closed states in OleP (e.g., the rupture/formation of salt bridges [26]) can shed some light to better describe such a mechanism at an atomic–molecular level.

**Figure 8:**
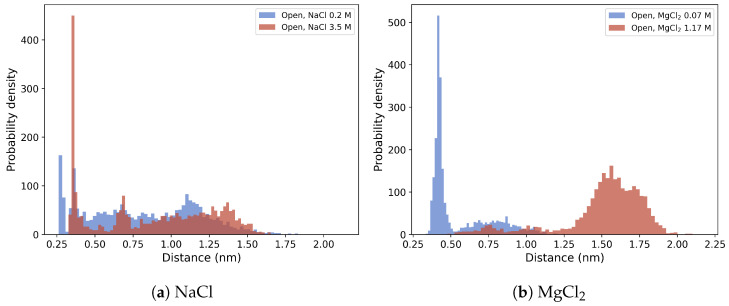
E233–R103 polar atoms side-chain distance distribution calculated in high (red) and low (blue) ionic strength in NaCl and MgCl_2_ buffer.

To this end, the electrostatic interactions between lateral ionic chains of specific residues, i.e., R168–E159–R123, E213–R116, K110–D227–R106 and E233–R103 (see Table S5), which break upon the open-to-closed conformational transition, were monitored along the MD trajectory of the open form in low- and high-ionic strength conditions for both the NaCl and MgCl_2_ buffers. The E223–R103 distance distribution, reported in Figure 8, clearly shows that the effect of the MgCl_2_ salt at 1.17 M is to space the two residues, as shown by the mode of the corresponding distribution located at ≈1.5 nm. Such an effect is not observed in NaCl or in MgCl_2_ at low ionic strength.

## 4. Discussion

In this work, we extend our previous studies on the ionic strength dependence of the heme-iron spin state and the conformational equilibrium of the cytochrome P450 OleP in complex with the aglycone substrate.

**Figure 9:**
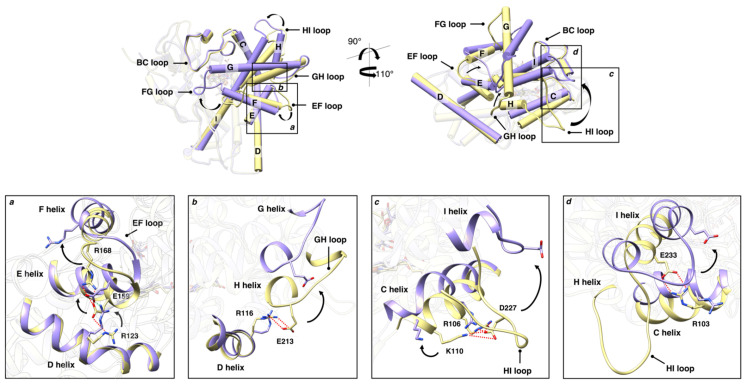
Putative ionic tethers in OleP. Top: open (yellow) and closed (purple) structures of OleP-6DEB complex are superposed and shown in different orientations, highlighting the secondary structure elements mainly involved in the conformational change. Helices are represented as cylinders. Squared black boxes, named from (**a**–**d**), indicate the regions where the possible ionic tethers identified in OleP are located. Bottom: details of individual regions (**a**–**d**) and amino acids involved in intramolecular electrostatic interactions are represented. Red and green dashed lines represent electrostatic interactions found in the open and left in the closed structures, respectively. In all panels, arrows indicate the direction of the open-to-closed transition.

The relationship between the electrolytic environment and the conformational transitions accompanying the heme-iron spin shift upon substrate binding has been interpreted for other P450s in the light of the ionic tethering mechanism [26]. According to this hypothesis, a pivotal contribution to the regulation of the substrate-binding process is provided by intramolecular electrostatic interactions between charged residues in the protein: the formation or rupture of these contacts may influence structural changes coupled to substrate binding. In P450s the ionic tethering mechanism has been described as an additional factor that regulates the substrate-binding process because it modulates the active site accessibility and the degree of hydration by boosting water expulsion from the heme pocket. This limits the production of reactive oxygen species, and ensures efficient coupling of monooxygenation [27,28,29,30,31]. Therefore, because the electrostatic properties of the environment may affect the strength of intramolecular salt links, the dependence of the structural transitions of a protein on the presence of electrolytes in solution, as is the case of OleP, points out on a conformational dynamics, which is under the ionic tethering control. Then, as described for other P450s [27,28,32], we may expect that an increase of ionic strength that stabilizes the closure of OleP (Figure 5 and Figure 6 and [5,7]), facilitating water expulsion from the active site (Figure 7b,c), and shifts the spin of the heme-iron to the high configuration (Figure 2 and Figure S3 and [7,11]), may cause the dissociation of some internal salt links of the protein. Searching for intramolecular electrostatic interactions that may act as ionic tethers in OleP, we compared the open and closed structures of OleP-6DEB, and we identified four ionic contacts over a total of 17, which are broken along the open-to-closed transition (Figure 9, Table S5).

These contacts are located on external regions of the protein, which participate in the structural transition, namely the EF, GH, and HI loops, and helices C and I. In more detail, we identified the electrostatic interactions involving (i) R168–E159–R123 in the region of the EF loop, and E and D helices, which break in the closed form, releasing the F helix and favoring its movement together with the connected FG unit toward the top of the OleP active site (Figure 9 box (a); (ii) E213–R116, respectively located on the GH loop and helix D, whose rupture upon the open-to-closed transition may accompany the motion of the connected helices, namely G and H (Figure 9 box (b); (iii–iv) K110–D227–R106 and R103–E233 that connect helix C with the region of the HI loop and helix I (Figure 9 boxes (c,d)). The disruption of these connections may contribute to the pronounced rearrangement of the HI loop that follows the I helix bending occurring upon the productive binding of the substrate. This movement sustains all the subsequent structural events that expand over the entire FG portion determining the closure of the OleP access channel [5].

On the basis of the above considerations combined to the identification of possible ionic tethers, we propose that the substrate-binding process of the P450 OleP is assisted by an electrostatic tethering mechanism. The conformational transition experienced by the enzyme upon substrate binding is accompanied by the dissociation of intramolecular electrostatic interactions confined to external sites of the protein, which ensures a fine regulation of the heme pocket accessibility and hydration.

To support this view of a contribution of ionic tethering to the regulation of OleP substrate binding, we investigated the conformational dynamics of OleP-6DEB complex, in both closed and open forms and in different ionic environments by means of MD simulations. Our analysis showed that in the high ionic strength conditions the closed conformation is favored. The principal component analysis on the MD trajectories and the Euclidean distances between P85 and T185 residues, located on the G helix and BCloop regions, respectively, indicate that high-ionic strength conditions shift the sampled conformations toward closed-like conformation. Similar observations can be drawn for the MD data obtained for the low- and high-ionic strength conditions in both NaCl and MgCl_2_ buffer. However, divalent cations cause a more pronounced stabilization of the closed conformations at high ionic strength. This is in line with the UV-visible spectral changes induced by substrate binding in different salts conditions, in which in the presence of divalent cations an enhanced accumulation of the high-spin population of OleP-6DEB in solution with respect to monovalent cations was observed.

Importantly, even if we found a general influence of ionic strength on the conformational dynamics of substrate-bound OleP, differences in the contribution to the process due to type of cation were also observed. Indeed, the analysis of the hydration structure of the active site of OleP-6DEB complex revealed that, in the presence of Na^+^, as representative for monovalent cations, and high-ionic strength environment, the expulsion of water molecules is the principal mechanism leading to a population shift toward the closed state. On the other hand, in the case of MgCl_2_ salt, the increase in the stabilization of closed-like conformations is due to an effect of divalent cations on the stability of the OleP ion tethers. In fact, under high-ionic strength conditions, MD simulations show a higher destabilization of the interaction between E233 carboxyl endpoint, located at the N-terminus of helix I, and guanidinio group of R103, on helix C, in the presence of Mg^2+^. The rupture of this interaction, which appears specifically promoted by the presence of divalent cations, might boost the conformational rearrangement of the HI loop and helix I, which is one of the main structural determinants of the OleP transition, in response to substrate binding [5]. Notably, we cannot exclude the possibility that the internal electrostatic interactions identified within the crystal structures might be differently distributed in solution, and that the effect of Mg^2+^ that we observed on a single charged-pair residue might extend to other intramolecular electrostatic contacts.

It is of interest to comment on the ion-tethering mechanism in the context of the natural substrate versatility of OleP. We already demonstrated that the intrinsic substrate promiscuity of OleP, which catalyzes its reaction on two structurally diverse metabolites [4], is sustained by water molecules that structure within the active site, establishing protein–substrate bridging interactions, to compensate for missing functional units of non-specific ligands [7]. Indeed, we observed that binding of the suboptimal aglycone substrate, such as 6DEB (which is a molecule that fits loosely the active site of OleP, thus preventing an efficient catalysis) leads to the accumulation of water in a cavity of the active site that forms upon OleP closure. In the presence of the preferred monoglycosylated substrate, this cavity would be fully occupied by the glycosyl moiety, thus favoring the complete removal of extra water molecules [5,7]. Previous works on the P450EryF (namely, 6-deoxyerythronolide B hydroxylase from *Saccharopolyspora erythraea*) and P450cam (namely, camphor 5-monooxygenase from *Pseudomonas putida*), in which the effect of salts on the P450 secondary structure and on substrate-induced spin shift was monitored by means of in-solution experiments and molecular dynamics simulations, have shown the electrostatic tethering to become more effective when the P450 binds nonoptimal substrates whose loose fit with the protein-binding pocket would lead to the accumulation of additional water molecules in the active site, resulting in a reduced enzymatic efficiency [27,28]. By analogy with those systems, it is reasonable to assume that an ion-tethering control of the heme pocket accessibility and hydration is required in a promiscuous P450 such as OleP, which shows broad substrate specificity, to maintain a discrete catalytic efficiency even in the presence of suboptimal substrates.

## 5. Conclusions

In summary, the results presented in this work, in the context of our previous analyses [5,7,11] and of data available in literature for other P450s [27,28], indicate that the ionic strength affects the conformational dynamics of the substrate-bound OleP through two main mechanisms: active site dehydration and ionic tether stability. A high concentration of NaCl, and most likely of other monovalent cations, mainly contributes to the process through the first route: they attract water molecules from the active site toward the protein surface, favoring the protein closure. In the presence of MgCl_2_, the hydration of the active site remains quite unaffected, but it induces the disruption of at least one of the salt bridges present in the open conformations, thus perturbing the strength of the ionic tethers. Therefore, our data corroborate the idea that the ionic tethering mechanism may play a pivotal role in the regulation of substrate and water accessibility of the active site in promiscuous cytochrome P450s, as a requirement of a higher level of control to prevent the accumulation of an excess of water molecules within the catalytic chamber, which could lead to harmful reactions.

## Figures and Tables

**Table 1:** Affinity constant and high-spin content for the binding of 6DEB to OleP in different salt conditions (I = 0.5 M).

	K_*D*_ (μM)	$\Delta G_{\mathrm{binding}}$ (kcal · mol^−1^)	$\Delta {\mathrm{Abs}}_{419/388}$	% HS (substrate-free)	% HS @ [6DEB] = 90 µM
No salt	3.83 ± 0.06	−7.39 ± 0.01	0.05/0.04	32	46
LiCl	2.89 ± 0.02	−7.55 ± 0.01	0.09/0.07	34	56
NaCl	5.43 ± 0.08	−7.18 ± 0.01	0.07/0.06	33	55
KCl	3.71 ± 0.03	−7.40 ± 0.01	0.07/0.06	32	55
NH_4_Cl	7.52 ± 0.05	−6.99 ± 0.01	0.08/0.06	33	53
MgCl_2_	3.18 ± 0.07	−7.50 ± 0.01	0.11/0.10	33	67
CaCl_2_	1.54 ± 0.02	−7.92 ± 0.01	0.11/0.09	38	67
SrCl_2_	2.82 ± 0.01	−7.57 ± 0.01	0.12/0.09	34	65

Salt concentrations were adjusted to achieve a final ionic strength of I = 0.5 M in all experiments. The K_D_ values were estimated from a global analysis of ∼50 different wavelengths. ΔAbs_419/388_ is the absorbance difference measured at 419 and 388 nm, respectively.

## Data Availability

Not applicable.

## Supplementary Materials

The following supporting information can be downloaded at: https://www.mdpi.com/article/10.3390/molecules28020832/s1.
