# Supplementary material for: Effect of Salts on the Conformational Dynamics of the Cytochrome P450 OleP
Source: Molecules. 2023 Jan 13;28(2):832. doi: 10.3390/molecules28020832 (PMC9867029; doi:10.3390/molecules28020832)
Supplement: Supplementary file 1 [file molecules-28-00832-s001.zip › template_supplementary.pdf]

molecules

MDPI

Article

Effect of Salts on the Conformational Dynamics of the Cytochrome P450 OleP

Maria Laura De Sciscio [1,†], Alessandro Nicola Nardi [1,†], Giacomo Parisi [2], Giovanni Bulfaro [3,4], Antonella Costanzo [3,4,5], Elena Gugole [3], Cécile Exertier [5], Ida Freda [3], Carmelinda Savino [5], Beatrice Vallone [3,*], Linda Celeste Montemiglio [5,*] and Marco D'Abramo [1,*]

1 Department of Chemistry, University of Rome, Sapienza, P.le A. Moro 5, 00185 Rome, Italy)
2 Center for Life Nano & Neuro-Science, Fondazione Istituto Italiano di Tecnologia, IIT, 00185 Rome, Italy
3 Department of Biochemical Sciences "A. Rossi Fanelli", University of Rome, Sapienza, P.le A. Moro 5, 00185 Rome, Italy)
4 Takis Biotech, Via di Castel Romano 100, 00128 Rome, Italy
5 Institute of Molecular Biology and Pathology, CNR c/o Department of Biochemical Sciences "A. Rossi Fanelli", University of Rome, Sapienza, P.le A. Moro 5, 00185 Rome, Italy
* Correspondence: beatrice.vallone@uniroma1.it (B.V.); lindaceleste.montemiglio@cnr.it (L.C.M.); marco.dabramo@uniroma1.it (M.D.)
† These authors contributed equally to this work.

check for updates

Citation: De Sciscio, M.L.; Nardi, A.N.; Parisi, G.; Bulfaro, G.; Costanzo, A.; Gugole, E.; Exertier, C.; Freda, I.; Savino, C.; Vallone, B.; et al. Effect of Salts on the Conformational Dynamics of the Cytochrome P450 OleP. *Molecules* **2023**, *1*, 0. https://doi.org/

Received: 13 December 2022
Revised: 10 January 2023
Accepted: 10 January 2023
Published: 13 January 2023

Copyright: © 2023 by the authors. Licensee MDPI, Basel, Switzerland. This article is an open access article distributed under the terms and conditions of the Creative Commons Attribution (CC BY) license (https://creativecommons.org/licenses/by/4.0/).

1. 6DEB Force Field Parameters

The following parameters for the 6-deoxyerythronolide B (6DEB) molecule were obtained by using CGenFF [1] in a form which is compatible with the CHARMM36 force field [2]. The obtained parameters were not further optimized.

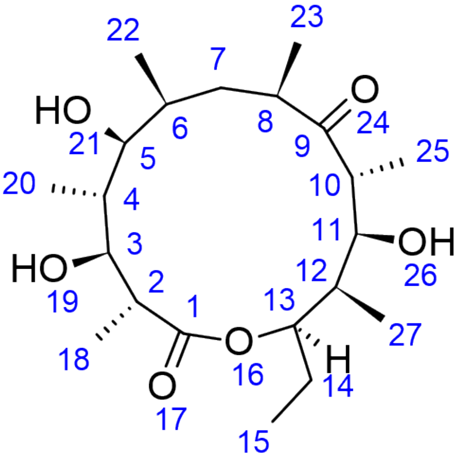

Figure S1. Indices of 6DEB nonhydrogen atoms.

In the following table, we report the list of the atom types used.

In Table S1 all the hydrogen atoms and their atom types are reported explicitly, but in Figure S1, for sake of clarity, the hydrogen atoms were not indicated. However, for methylic, methylenic and methynic hydrogen atoms the HGA3, HGA2, and HGA1 atom types were used, respectively. For the hydroxilic hydrogen atoms the HGP1 atom type was used.

In the following tables, we report the list of the additional bond parameters used, in addition to the ones already present in CHARMM36 force field.

Table S1. Atom types, masses and atomic charges used for the parameterization of 6DEB. The atom indices refer to the Figure S1.

Index	Atom Name	Atom Type	Mass (Da)	Charge (e)	σ (nm)	ϵ (kJ/mol)
1	C1	CG2O2	12.011	0.887	0.302905564168	0.41003
2	C2	CG311	12.011	−0.117	0.356359487256	0.13389
3	C3	CG311	12.011	0.130	0.356359487256	0.13389
4	C4	CG311	12.011	−0.082	0.356359487256	0.13389
5	C5	CG311	12.011	0.138	0.356359487256	0.13389
6	C6	CG311	12.011	−0.076	0.356359487256	0.13389
7	C7	CG321	12.011	−0.180	0.358141284692	0.23430
8	C8	CG311	12.011	−0.002	0.356359487256	0.13389
9	C9	CG2O5	12.011	0.283	0.356359487256	0.37656
10	C10	CG311	12.011	0.005	0.356359487256	0.13389
11	C11	CG311	12.011	0.145	0.356359487256	0.13389
12	C12	CG311	12.011	−0.089	0.356359487256	0.13389
13	C13	CG311	12.011	0.176	0.356359487256	0.13389
14	C14	CG321	12.011	−0.193	0.358141284692	0.23430
15	C15	CG331	12.011	−0.258	0.365268474438	0.32635
17	O17	OG2D1	15.999	−0.634	0.302905564168	0.50208
18	C18	CG331	12.011	−0.270	0.365268474438	0.32635
20	C20	CG331	12.011	−0.269	0.365268474438	0.32635
22	C22	CG331	12.011	−0.271	0.365268474438	0.32635
23	C23	CG331	12.011	−0.261	0.365268474438	0.32635
24	O24	OG2D3	15.999	−0.501	0.302905564168	0.20920
25	C25	CG331	12.011	−0.259	0.365268474438	0.32635
27	C27	CG331	12.011	−0.269	0.365268474438	0.32635
28	H1	HGA1	1.008	0.090	0.238760856462	0.18828
29	H2	HGA1	1.008	0.090	0.238760856462	0.18828
30	H3	HGA1	1.008	0.090	0.238760856462	0.18828
31	H4	HGA1	1.008	0.090	0.238760856462	0.18828
32	H5	HGA1	1.008	0.090	0.238760856462	0.18828
33	H6	HGA2	1.008	0.090	0.238760856462	0.14644
34	H7	HGA2	1.008	0.090	0.238760856462	0.14644
35	H8	HGA1	1.008	0.090	0.238760856462	0.18828
36	H9	HGA1	1.008	0.090	0.238760856462	0.18828
37	H10	HGA1	1.008	0.090	0.238760856462	0.18828
38	H11	HGA1	1.008	0.090	0.238760856462	0.18828
39	H12	HGA1	1.008	0.090	0.238760856462	0.18828
40	H13	HGA2	1.008	0.090	0.238760856462	0.14644
41	H14	HGA2	1.008	0.090	0.238760856462	0.14644
42	H15	HGA3	1.008	0.090	0.238760856462	0.10042
43	H16	HGA3	1.008	0.090	0.238760856462	0.10042
44	H17	HGA3	1.008	0.090	0.238760856462	0.10042
45	H18	HGA3	1.008	0.090	0.238760856462	0.10042
46	H19	HGA3	1.008	0.090	0.238760856462	0.10042
47	H20	HGA3	1.008	0.090	0.238760856462	0.10042
48	H21	HGP1	1.008	0.419	0.040001352445	0.19246
49	H22	HGA3	1.008	0.090	0.238760856462	0.10042
50	H23	HGA3	1.008	0.090	0.238760856462	0.10042
51	H24	HGA3	1.008	0.090	0.238760856462	0.10042
52	H25	HGP1	1.008	0.419	0.040001352445	0.19246
53	H26	HGA3	1.008	0.090	0.238760856462	0.10042
54	H27	HGA3	1.008	0.090	0.238760856462	0.10042
55	H28	HGA3	1.008	0.090	0.238760856462	0.10042
56	H29	HGA3	1.008	0.090	0.238760856462	0.10042
57	H30	HGA3	1.008	0.090	0.238760856462	0.10042
58	H31	HGA3	1.008	0.090	0.238760856462	0.10042
59	H32	HGA3	1.008	0.090	0.238760856462	0.10042
60	H33	HGA3	1.008	0.090	0.238760856462	0.10042
61	H34	HGA3	1.008	0.090	0.238760856462	0.10042
62	H35	HGP1	1.008	0.419	0.040001352445	0.19246
63	H36	HGA3	1.008	0.090	0.238760856462	0.10042
64	H37	HGA3	1.008	0.090	0.238760856462	0.10042
65	H38	HGA3	1.008	0.090	0.238760856462	0.10042

Table S2. Angle bend parameters.

Atom Type	Atom Type	Atom Type	θ_0 (deg.)	k_θ (kJ/mol)
CG311	CG2O5	CG311	115.600000	292.880000
CG2O5	CG311	CG311	108.000000	435.136000
CG2O5	CG311	CG321	108.000000	435.136000
CG2O5	CG311	CG331	108.000000	435.136000
CG311	CG311	OG302	109.700000	962.320000

Table S3. Proper dihedral angle parameters.

Atom Type	Atom Type	Atom Type	Atom Type	ϕ_0 (deg.)	k_ϕ (kJ/mol)	mult.
CG311	CG2O2	OG302	CG311	180.000000	8.577200	2
CG311	CG2O5	CG311	CG311	0.000000	3.138000	1
CG311	CG2O5	CG311	CG311	180.000000	0.753120	2
CG311	CG2O5	CG311	CG311	0.000000	0.271960	3
CG311	CG2O5	CG311	CG311	0.000000	0.125520	6
CG311	CG2O5	CG311	CG321	0.000000	3.138000	1
CG311	CG2O5	CG311	CG321	180.000000	0.753120	2
CG311	CG2O5	CG311	CG321	0.000000	0.271960	3
CG311	CG2O5	CG311	CG321	0.000000	0.125520	6
CG311	CG2O5	CG311	CG331	0.000000	3.138000	1
CG311	CG2O5	CG311	CG331	180.000000	0.753120	2
CG311	CG2O5	CG311	CG331	0.000000	0.271960	3
CG311	CG2O5	CG311	CG331	0.000000	0.125520	6
CG311	CG2O5	CG311	HGA1	0.000000	0.418400	3
OG2D3	CG2O5	CG311	CG311	180.000000	3.138000	1
OG2D3	CG2O5	CG311	CG311	180.000000	0.753120	2
OG2D3	CG2O5	CG311	CG311	180.000000	0.271960	3
OG2D3	CG2O5	CG311	CG311	0.000000	0.125520	6
OG2D3	CG2O5	CG311	CG321	180.000000	3.138000	1
OG2D3	CG2O5	CG311	CG321	180.000000	0.753120	2
OG2D3	CG2O5	CG311	CG321	180.000000	0.271960	3
OG2D3	CG2O5	CG311	CG321	0.000000	0.125520	6
OG2D3	CG2O5	CG311	CG331	180.000000	3.138000	1
OG2D3	CG2O5	CG311	CG331	180.000000	0.753120	2
OG2D3	CG2O5	CG311	CG331	180.000000	0.271960	3
OG2D3	CG2O5	CG311	CG331	0.000000	0.125520	6
CG2O2	CG311	CG311	CG311	0.000000	0.836800	3
CG2O5	CG311	CG311	CG311	0.000000	0.836800	3
CG2O5	CG311	CG311	OG311	0.000000	0.836800	3
CG2O5	CG311	CG311	HGA1	0.000000	0.836800	3
CG311	CG311	CG311	CG311	180.000000	2.092000	4
CG311	CG311	CG311	OG302	180.000000	0.836800	3
CG321	CG311	CG311	CG331	180.000000	2.092000	4
CG331	CG311	CG311	OG302	0.000000	0.836800	3
OG302	CG311	CG311	HGA1	0.000000	0.815880	3
CG2O5	CG311	CG321	CG311	0.000000	0.836800	3
CG2O5	CG311	CG321	HGA2	0.000000	0.836800	3
OG302	CG311	CG321	CG331	180.000000	0.669440	1
OG302	CG311	CG321	CG331	0.000000	1.631760	2
CG2O5	CG311	CG331	HGA3	0.000000	0.836800	3
CG311	CG311	OG302	CG2O2	180.000000	2.928800	1

Table S4. Improper dihedrals parameters.

Atom Type	Atom Type	Atom Type	Atom Type	ϕ_0 (deg.)	k_ϕ (kJ/mol)
CG2O5	CG311	CG311	OG2D3	0.000000	585.760000

2. Equilibration Phase of Molecular Dynamics Simulations

For the equilibration phase, we performed a 50-ps MD simulation, then the mean pressure was calculated along the trajectory. If the pressure is lower/higher than the value of 560 bar, we decrease/increase the size of the box by 0.03 nm. With the new box size, we run a new 50-ps MD simulation, providing as the initial positions the last frame of the previous run. We continue this scheme until the mean value of the pressure along the n-th 50-ps MD simulation reaches (560 $\pm$ 30) bar. This value of the pressure was determined

in a previous work [3] to correctly reproduce the solvent bulk density and the protein hydration shell. For the present study, twenty 50-ps equilibration simulations, for each system, were run.

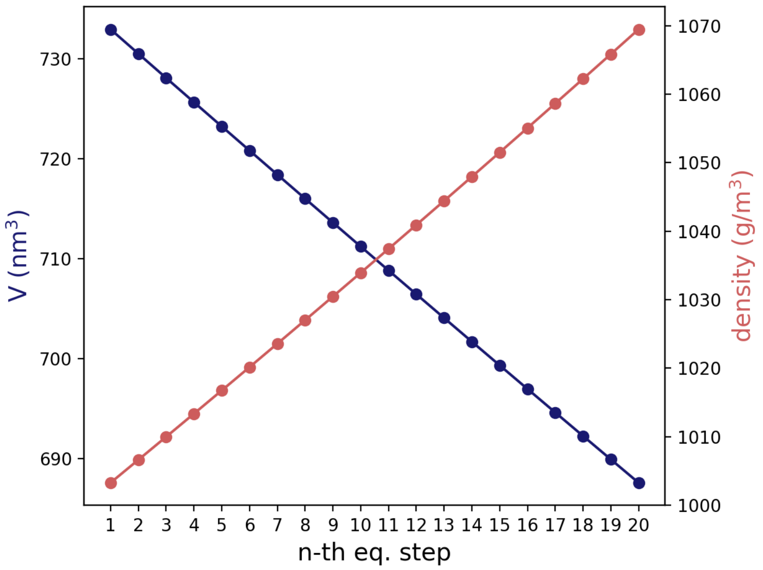

Figure S2. Volume and density of the simulated box as a function of the n-th 50 ps short MD equilibration simulation.

3. Cation-Dependent Effect on the Spin-State Equilibrium of OleP-6DEB

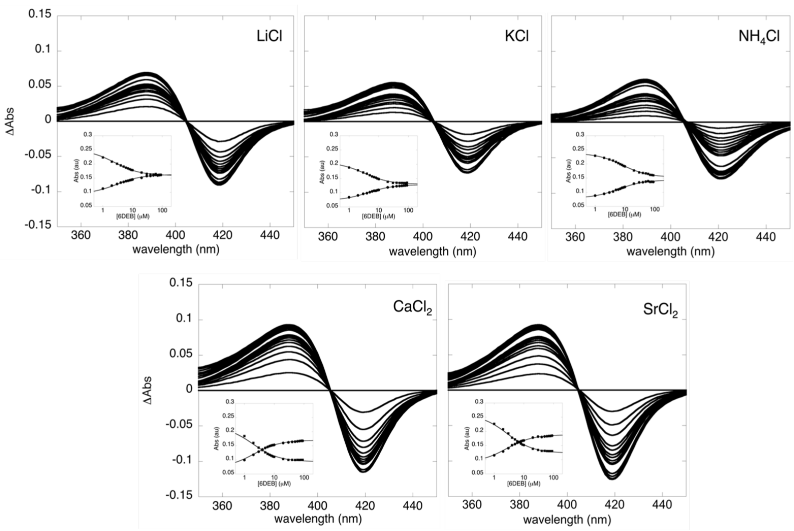

Figure S3. Equilibrium titrations of OleP with 6DEB in 50-mM Hepes, pH 7.5, 298 K, in the presence of different mono- and divalent cations at I = 0.5 M. Difference spectral changes observed upon substrate binding are shown. Insets report the absorbance intensities of OleP monitored at 419 nm (full squares) and at 388 nm (full dots) as a function of the logarithm of total 6DEB concentration. Solid lines are the best fit to a hyperbolic function.

4. Intramolecular Electrostatic Interactions Identified in the Open Form of Substrate Bound OleP

Table S5. Intramolecular electrostatic interactions identified in the open form of substrate bound OleP.

Interaction	Location	Rupture Upon the Open-to-Close Transition
R35–E328	A helix–K′L loop	no
R42–E315	β strand β1–β hairpins β3	no
R57–D327	β strand β2 - K′ helix	no
R103–E233	C helix–I helix	yes
R105-–heme propionate	C helix–heme	no
R106–D227–K110	C helix–HI loop–C helix	yes
R116–E213	D helix–GH loop	yes
R121–D118–R125–E368	D helix–D helix–D helix–L helix	no
R123–E159–R168	D helix–E helix–EF loop	yes
R207–D214	G helix–GH loop	no
K231–D201	I helix—helix G	no
R286–E283–R341	K helix–K helix–K′L loop	no
R298–heme propionate	β hairpins 3–heme	no
R363–E282	L helix–K helix	no
R375–D129	L helix–D helix	no
R376–D133	L helix–D helix	no
R402–E384	β hairpins β4–β hairpins β4	no

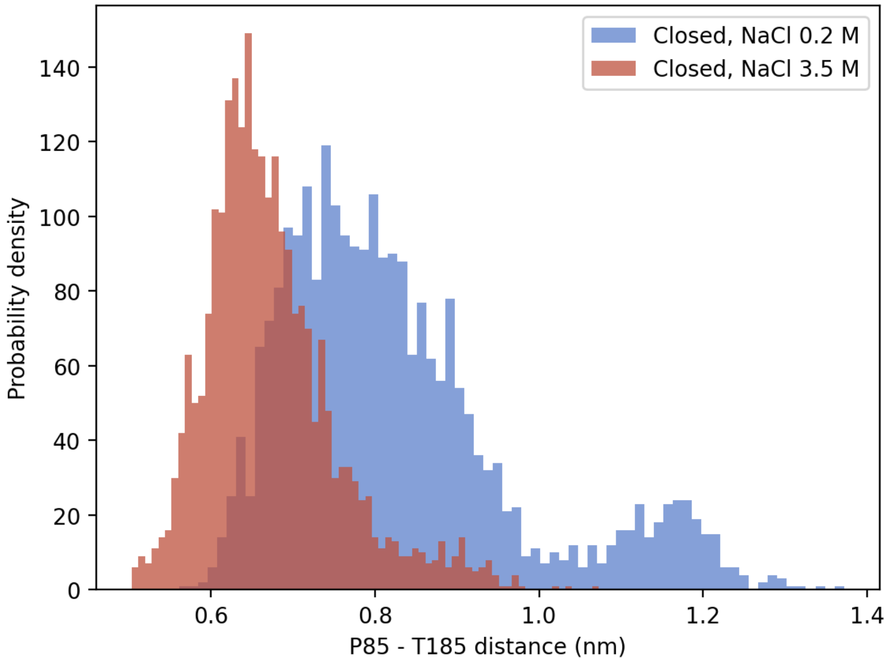

Figure S4. P85–T185 distance distribution, descriptor of OleP open-to-close transition, for OleP closed form in high (red) and low (blue) ionic strength in NaCl buffer.

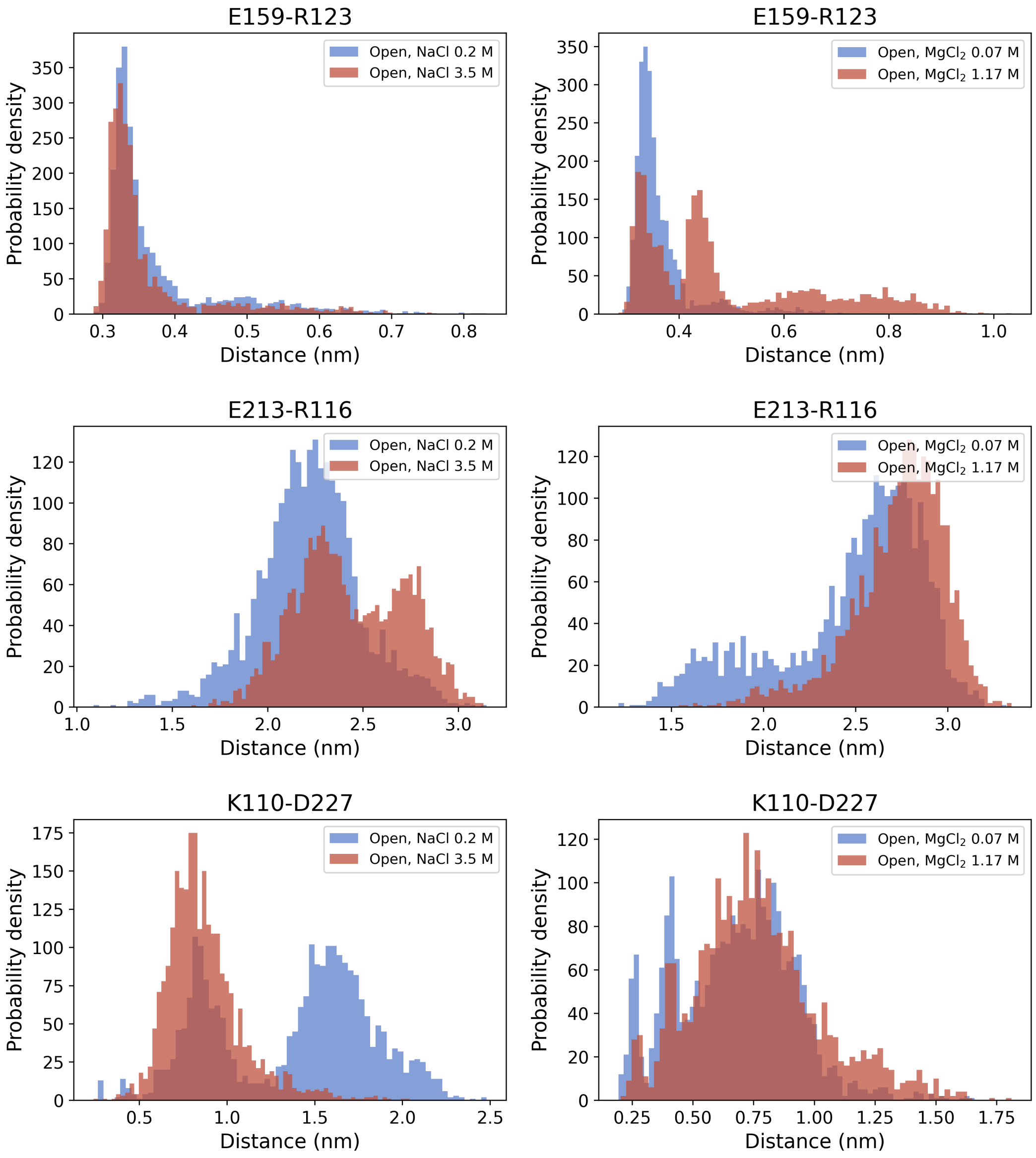

(**a**) NaCl (**b**) $MgCl_2$

Figure S5. Electrostatic interactions between side-chain ion group of E159–R123, E213–R116 and K110–D227 residues in high (red) and low (blue) ionic strength condition in NaCl (**a**) and $MgCl_2$ (**b**) buffer.

References

1. Soteras Gutiérrez, I.; Lin, F.Y.; Vanommeslaeghe, K.; Lemkul, J.A.; Armacost, K.A.; Brooks, C.L.; MacKerell, A.D. Parametrization of halogen bonds in the CHARMM general force field: Improved treatment of ligand–protein interactions. *Bioorg. Med. Chem.* **2016**, *24*, 4812–4825.
2. Best, R.B.; Zhu, X.; Shim, J.; Lopes, P.E.M.; Mittal, J.; Feig, M.; MacKerell, A.D.J. Optimization of the Additive CHARMM All-Atom Protein Force Field Targeting Improved Sampling of the Backbone φ, ψ and Side-Chain $\chi1$ and $\chi2$ Dihedral Angles. *J. Chem. Theory Comput.* **2012**, *8*, 3257–3273.
3. Del Galdo, S.; Marracino, P.; D'Abramo, M.; Amadei, A. In silico characterization of protein partial molecular volumes and hydration shells. *Phys. Chem. Chem. Phys.* **2015**, *17*, 31270–31277.

Disclaimer/Publisher's Note: The statements, opinions and data contained in all publications are solely those of the individual author(s) and contributor(s) and not of MDPI and/or the editor(s). MDPI and/or the editor(s) disclaim responsibility for any injury to people or property resulting from any ideas, methods, instructions or products referred to in the content.
